# Exploring the Nexus Between Mindfulness, Gratitude, and Wellbeing Among Youth With the Mediating Role of Hopefulness: A South Asian Perspective

**DOI:** 10.3389/fpsyg.2022.915667

**Published:** 2022-07-19

**Authors:** Salima Barkat Ali, Munawar Javed Ahmad, Iqra Ramzan, Muhammad Ali, Kalimullah Khan

**Affiliations:** ^1^Department of Psychology, Iqra University, Karachi, Pakistan; ^2^Department of Business Administration, Hazara University, Mansehra, Pakistan; ^3^Department of Business Administration, Kardan University, Kabul, Afghanistan

**Keywords:** mindfulness, gratitude, wellbeing, hopefulness, Pakistan

## Abstract

This study investigates the relationship between mindfulness, gratitude, and psychological wellbeing of young individuals in Pakistan with the potential role of hopefulness as a mediator between mindfulness, gratitude, and wellbeing. Data were collected from young individuals (18–40 years old) from Pakistan. A total sample of 500 participants was collected by employing the online survey questionnaire, and 374 questionnaires were duly filled and returned. The PLS-SEM technique was used to test the proposed hypotheses. The results of the study found that there is a strong direct relationship between gratitude, mindfulness, and hopefulness, and mindfulness is also strongly correlated with wellbeing. However, the relationship between gratitude and wellbeing was not statistically significant. Moreover, the mediation results reveal that the relationship between mindfulness, gratitude, and wellbeing is significantly mediated by hopefulness. This shows that gratitude and mindfulness are crucial in enhancing wellbeing through hopefulness. This study is an important contribution to validating the broaden-and-build theory, which suggests that hopelessness is a significant factor of a depressive state. It can be indicated that inducing hopefulness could be a significant element of the treatment plan of professional clinical psychologists.

## Introduction

For the past 2 years, the epidemic of COVID-19 has affected the daily functioning of human beings. It has given rise to traumas, depression, fear, despair, and anxiety (Ahorsu et al., 2020), which are closely related to the overall life satisfaction and wellbeing of an individual (Headey et al., 1993; Mukhtar, 2020). In addition, mental health services worldwide have been significantly affected by COVID-19, which is one of the reasons for the increase in psychological disturbances. According to the World Health Organization (WHO), the COVID-19 epidemic has crippled or, in some cases, hampered vital psychological health services in 93% of countries worldwide, increasing the demand for mental health.

The current epidemic also highlighted some of the causes of mental illness, such as global inequality in attaining healthcare due to race and ethnicity, gender, and lack of respect to human rights, especially for those living with mental disorders (Saleem, 2021). Lack of investment in mental health further contributes to the increase in mental health problems in people. The International Labor Organization (ILO) surveyed more than 12,000 individuals from 112 countries at the end of 2020. The results showed that unemployment and job losses are positively correlated with youth anxiety and depression.

As 63% of the Pakistani population is youth, this is more alarming for Pakistan. Approximately 50 million people in Pakistan have psychological disorders, as reported before the pandemic (Saleem, 2021), and now, the situation is much worse. According to data released in the Economic Survey 2021, Pakistan is spending far less on healthcare than the World Health Organization (WHO) recommended; the spending is 1.2% of GDP on healthcare compared to the WHO's recommendation, which is 5%. This is another sign that mental health problems will increase in the coming years.

Sindh Mental Health Authority (SMHA) and Edhi Foundation conducted a survey in Pakistan during COVID-19 which showed that 42% of participants reported suffering from depression, high levels of anxiety, socioemotional problems, and 25% experiencing suicidal thoughts (Yusuf, 2021). Similarly, the WHO announced that the mental health effects of the epidemic would be “long-term and far-reaching.” As the world is locked down, such a conversation is not novel, government and health officials acknowledged that fear of illness, disruption of work, limited social interaction, and increased family tensions would have psychological and emotional consequences. The SMHA survey tried to explain this understanding; findings suggested that 62% of respondents pinpoint pandemic-linked loss of income, and 72% pinpoint food insecurity as the primary driver of their depression. It has been suggested that political instability and climate change will be more likely to intensify these drivers. The mental health challenges will pile up the present distress.

The statistics mentioned indicates the severity of Pakistan's mental health crises, which needs to address. Notably, post-pandemic mental health crises in Pakistan need to study due to increasing mental health issues in Pakistan. However, studies have been conducted to understand the drivers of mental wellbeing, for instance, isolation, illnesses, anxiety, stress, social distancing, and pandemic (Mukhtar, 2020), positive emotion, subjective wellbeing (SWB), psychological wellbeing (PWB), and inner harmony (Corey, 2002; Delle Fave et al., 2016). At the same time, this study focuses on the post-pandemic situation to investigate the mental wellbeing among the individuals of Pakistan. Emerging studies have reported that gratitude, mindfulness, and hopefulness are the promising positive psychological tools that help an individual to cope with anxiety and stress and enhance psychological health, and wellbeing (O'Leary and Dockray, 2015; Omidi and Zargar, 2015; David, 2019; Pellerin and Raufaste, 2020). Considering the previous evidence, this study attempted to investigate the relationship between gratitude and mindfulness on subjective wellbeing (Mental wellbeing) with the mediating role of hopefulness.

As clinical psychologists and researchers have endorsed the importance of using positive psychology techniques in treating many psychological disorders (Bolier et al., 2013; Hanson, 2019; Özdemir and Kavak Budak, 2021) and enhancing wellbeing to decrease psychopathology (Grant et al., 2013; Lamers et al., 2015; Trompetter et al., 2017), specifically, mindfulness and gratitude have been found to positively impact positive mental wellbeing (David, 2019; Pellerin and Raufaste, 2020), but there is little research found in explaining the mechanism behind this construct. Therefore, this study has been designed to address this potential gap by studying hopefulness as a mediator between the relationship of gratitude, mindfulness, and wellbeing.

## Theoretical Framework and Hypothesis Development

This study has been conceptualized through broaden-and-built theory. It states that individuals with positive emotions tend to think and act in a more positive and healthy way, thus broadening the thought–action repertoire. Positive emotions like curiosity lead an individual to explore, openness to new experiences, and grow self to their maximum (Fredrickson, 1998). Fredrickson (2001) further suggested that these emotions enhance an individual's coping resources, including knowledge and social networks. Garland et al. (2010) supported the view that positive emotions have the power to influence the action repertoires and resilience positively. Fredrickson (2001) believed that broadening and building processes of emotions enhance subjective wellbeing in adults, as it is also evidenced that positive emotions are more likely to predict positive indicators in life satisfaction (Datu and King, 2016).

Aligning with this theory, hopefulness serves as a positive emotion (Fredrickson et al., 2003), so it can be suggested that hopefulness encompasses the two processes, personal resilience and thought–action repertoires, of broaden-and-built theory. Personal resilience is implicated in the form of agency thinking and thought–action repertoires in pathway thinking (Chang et al., 2019). Hopefulness serves in broadening and building processes as it is considered as a positive emotion and affects the wellbeing of individuals (Chang et al., 2019).

**Figure 1:**
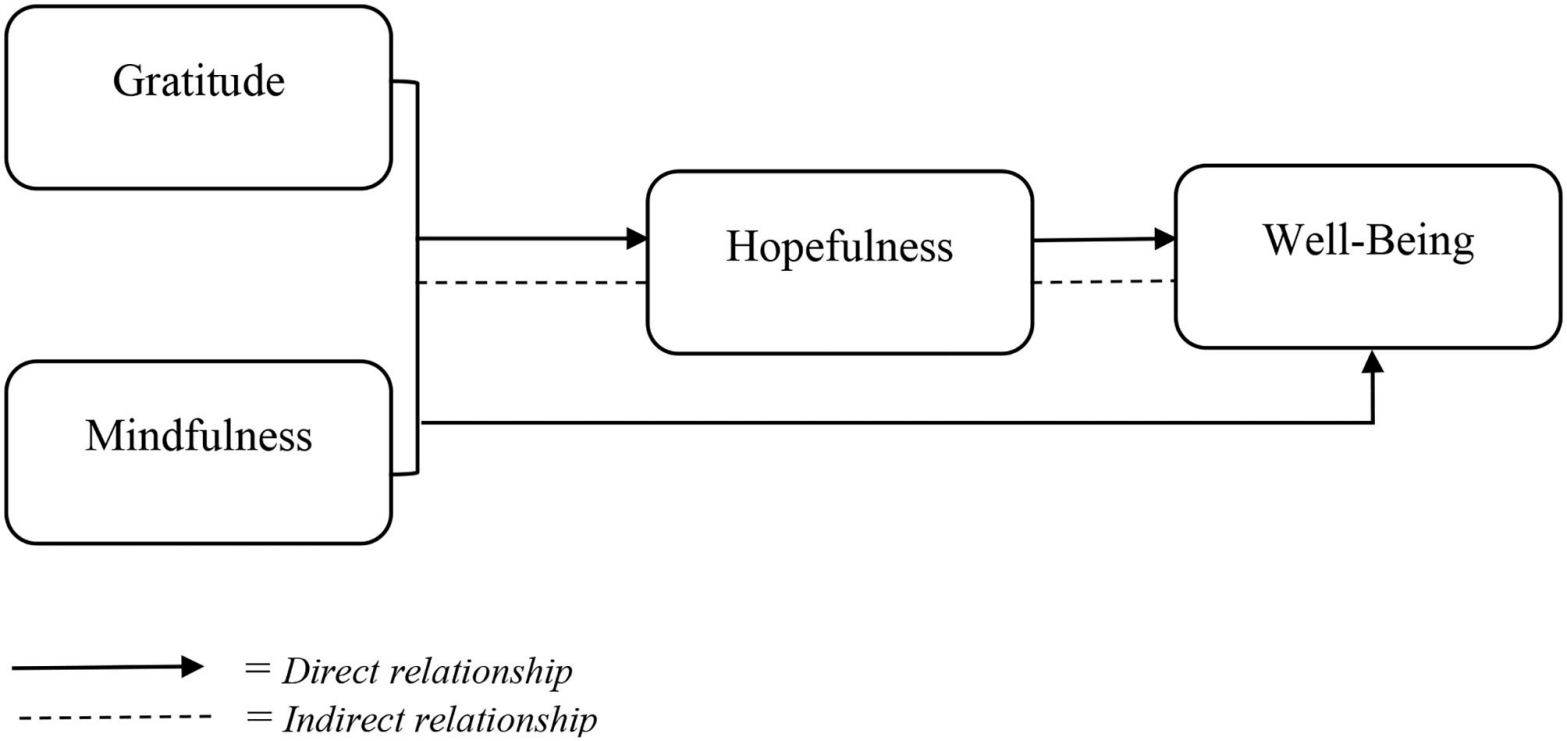
Research framework.

Gratitude and mindfulness promote a positive emotional state and treat daily stressors more effectively, and hence may enhance hopefulness resulting in explaining the process of mindfulness and gratitude with wellbeing. Researches provide extensive evidence for the broadening and building role of gratitude (e.g., Fredrickson, 2004; Wood and Tarrier, 2010). Along with these findings, we hypothesized that mindfulness, literature quoted as an emotional regulator (Brown et al., 2007), will help individuals to be more attentive to positivity within themselves and around them, resulting in high hope for future. In addition, this hopefulness will ultimately increase the wellbeing of individuals. Hence, we have proposed that gratitude and mindfulness will broaden and build other positive emotions and actions which will enhance wellbeing (Figure 1).

### Wellbeing

According to Carr (2013), the positive psychology approach majorly deals with the issues like wellbeing, happiness, and activities that enhance an individual's growth and develop meaningful relationships. In this context, wellbeing is studied regarding the positive side of an individual's life. Wellbeing, as defined by APA, is “a state of happiness and contentment, with low levels of distress, overall good physical and mental health and outlook, or good quality of life.”

It is widely credited that wellbeing is a multifaceted concept that includes multiple domains of human functioning. Wellbeing has been subdivided into subjective, objective, social, psychological, physical, and community wellbeing (Wilkinson, 1979; Kashdan et al., 2008; Burke et al., 2010; Pontin et al., 2013). In this study, they are being used as subjective wellbeing as a subjective experience. This study has taken physical, psychological, and relational wellbeing into account and used it as overall wellbeing. According to Pontin et al. (2013), wellbeing is divided into objective and subjective experiences of an individual. They have incorporated constructs of both eudemonic and hedonic theories into one theoretical framework and established the BBC subjective wellbeing scale. We have also constructed a present study's framework on this recent study and took psychological, physical, and relational wellbeing into account of subjective experience of wellbeing.

### Mindfulness

Mindfulness is explained as one's mental ability to keep self-attentive by paying attention intentionally (Park et al., 2013). In addition, mindfulness is the attention and awareness that is a universal ability inherent in human beings, and it is to pay attention in a special way, intentionally, at the present moment, thereby being aware of circumstances around the person in a non-judgmental way (Kabat-Zinn et al., 2011). Multiple factors are related to wellbeing. One of them is mindfulness. Researchers have studied mindfulness as a psychological construct and an emotional regulator in the previous few decades in the field of clinical psychology (Brown et al., 2007). In particular, mindfulness is studied as a predictor of better mental health. It has been used to treat mental health disorders such as anxiety, eating disorder, depression, sexual disorders, attention deficit hyperactivity, stress management, and substance abuse (Grossman et al., 2004; Chambers et al., 2009).

Recent studies have identified the connection of the above-mentioned features of mindfulness with self-determination (autonomy), personal growth, understanding others (positive relation with others), purpose in life, acceptance of self, and balance between inner and outer self (environmental mastery) (Wallace and Shapiro, 2006). Schutte and Malouff (2011) also suggested that higher life satisfaction, emotional intelligence, and positive effect and lower negative effect are the influencers of wellbeing. Hence, it can be said that mindfulness plays a vital role in one's positive mental health.

H1: Mindfulness has a direct effect on wellbeing.

### Gratitude

Gratitude is another characteristic which influences an individual's wellbeing. It has been conceptualized as an emotion, attitude, coping strategy, and trait or disposition (Emmons and Shelton, 2002; Emmons et al., 2003; David, 2019). According to the emotion paradigm, it has been explained as an emotion directly related to receiving support from others (McCullough et al., 2001). Thus, it is a positive feeling relating to the point of view of taking benefit from the actions of another individual (Emmons and Shelton, 2002; Emmons et al., 2003). In this perspective, gratitude serves as a motivator of helping and altruistic acts which result from receiving support from others. On the contrary, Abramson et al. (1978) explained gratitude in a more generalized sense of gratefulness for various characteristics of life, rather than taking it as social emotion. The tendency to be appreciative and grateful for the positive characteristics of life is called dispositional or trait gratitude. Moreover, Wood and Tarrier (2010) reported that gratitude is different from other positive emotions such as optimism and hope, and these positive emotions are explained by productive future expectations and the ability to identify the creative ways through which it can be achieved (Tong et al., 2010). In contrast, gratitude does not include expectations or other future-based thoughts. Instead, gratitude is primarily a definition of appreciating the positive facets of life in the present.

Looking from these different perspectives, it can be understood that gratitude is simply appreciation of the positive facets of the present life which may or may not need support from others. On the basis of this, this study operationalized gratitude as “the appreciation of what is valuable and meaningful to oneself and represents a general state of thankfulness and/or appreciation” (Sansone and Sansone, 2010). Gratitude is also correlated with depression. Wood et al. (2008) reported that having the feelings of thankfulness will make one's depressive phase more tolerable and shorter. Another study suggested that gratitude is negatively associated with depression (Seligman et al., 2005). According to the literature, individuals suffering from depression tend to have low levels of psychological wellbeing (e.g., Liu et al., 2009). Thus, on the basis of previous studies, it can be concluded that by increasing gratitude, psychological wellbeing can be enhanced.

H2: Gratitude has a direct effect on wellbeing.

### Hopefulness as Mediator

Hope is defined by Snyder (2002) as basic cognitive, aim-oriented ways of thinking in which an individual comes up with alternatives to attain their objectives and remains motivated to follow and amend these alternatives if needed. According to him, individuals having hope will be active and consistent in attaining their goals. The most comprehensive theory for hope is given by Snyder, proposing hope as a cognitive set having components: agency and pathway thinking (Snyder et al., 1991). Positive emotions and thinking, as explained in a theoretical framework, will enhance an individual's ability to broaden their expectancies for the future, increasing hopefulness and psychological adjustment in the environment (i.e., life satisfaction and positive affectivity; Watson and Naragon, 2009; Datu and King, 2016). Hope makes an individual feel happy and well, which ultimately increases the hopefulness for the future. It is directly associated with subjective wellbeing (Bailey and Snyder, 2007).

The construct of hopefulness and happiness is multidimensional and is explained differently by different disciplines and used in distinct modes in daily functioning (Webb, 2007). Generally, hopefulness is taken as a positive construct that helps an individual to have a favorable view of the future. Many psychologists take it more as a positive emotion that helps to cope with stressful life situations (Fredrickson et al., 2003). Hopefulness is positively correlated with good mental health and better daily life functioning. It has been seen that gratitude helps manage depressed feelings by enhancing psychological resources, that is, hope (Fredrickson, 2001). Hopefulness promotes positive expectation in future of the expected results (Scioli et al., 2011). Existing literature supports the positive link between hopefulness and better psychological health (e.g., Bailey and Snyder, 2007; Muyan-Yilik and Demir, 2020). Hopefulness significantly impacts life satisfaction, and negative and positive effects (Muyan-Yilik and Demir, 2020). Furthermore, mindful individuals tend to respond to the stressful situations more proactively, which means having hopefulness, optimism, self-efficacy, and resiliency, increasing the positive outlook toward the future (Malinowski and Lim, 2015). From the above literature, it can be suggested that hopefulness plays an important role in having the positive personal coping reservoir that directly relates to an individual's wellbeing.

Literature provides us evidence that gratitude and mindfulness have a unique effect on wellbeing (e.g., psychological and subjective; Fredrickson, 2001; Wood and Tarrier, 2010) but provides little about the process between these variables. This study aimed to expand the existing literature and narrow the gap of mediating effect of hopefulness in explaining the relationship between mindfulness and gratitude with wellbeing. To our knowledge, no prior research has explored the process through which these variables work. Thus, this study is the first in identifying hopefulness as a mediating factor of gratitude and mindfulness in explaining positive wellbeing. Moreover, this study also explores the relationship between mindfulness and gratitude. It has been reported by the previous studies that a strong association exists between gratitude and mindfulness (Swickert et al., 2019). This type of research will help mental health professionals to make strategies and interventions for enhancing the mental health of the Pakistani population specifically.

H3. Mindfulness has a direct effect on hopefulness.
H4. Gratitude has a direct effect on hopefulness.
H5. Hopefulness would mediate the relationship between gratitude and wellbeing.
H6. Hopefulness would mediate the relationship between mindfulness and wellbeing.

## Methodology

### Participants and Procedure

The cross-sectional quantitative method was used in this study. Adults ranging from 18 to 40 years of age were contacted as participants for this study. Self-administered questionnaires were used to obtain data from respondents. The respondents came from various socioeconomic backgrounds in terms of gender, marital status, education, family structure, and monthly income. The survey was entirely optional, and participants were informed that their information would be kept private and anonymous. A convenience sampling strategy was used based on previous research (Etikan et al., 2016). A total of 500 people were contacted and invited to participate in the survey. With a significance level of 5%, the statistical power of 80%, the Four constructs employed in this investigation, 47 items, and an effect size of 0.15, the research model required just 119 sample observations to discover R^2^ values of at least 0.25 (Hair et al., 2017). There were 374 valid responses in total. According to the results of the retrospective test (Cohen, 1992), recommended by statistical power analysis (Brysbaert, 2019), this number was sufficient. The sample has a statistical power of 0.95, which is higher than the suggested minimum of 0.80, according to G^*^ 3.1.9.2 software (Cohen, 1992).

### Measures

#### Gratitude

The Gratitude Questionnaire-Six-Item Form (GQ-6) was used to measure gratitude. It is a self-report questionnaire designed to assess individual differences in the proneness to experience gratitude in daily life. Participants rated their agreement with statements (e.g., “I am grateful to a wide variety of people”) using a five-point Likert-type scale from 1 (strongly disagree) to 5 (strongly agree).

#### Mindfulness

Mindfulness was measured through the Cognitive and Affective Mindfulness Scale-Revised (CAMS-R). The scale consists of 10 items (e.g., “I can accept things I cannot change.”) rated on a five-point Likert scale.

#### Wellbeing

The BBC subjective wellbeing scale (BBC-SWB), designed to measure people's subjective experiences across the wide breadth of domains commonly included in definitions of wellbeing, was used in this study to measure subjective wellbeing. The BBC-SWB comprised 24 items measured through a five-point Likert scale.

#### Hopefulness

To measure hopefulness, this study used The Adult Dispositional Hope Scale adopted by Snyder et al. (1991). This scale consists of 12 items (e.g., “I can think of many ways to get out of a jam”) measured through a five-point Likert scale.

### Analysis and Results

A structural equation model (SEM) investigated the relationship between constructs. Because of the complexity of the developed model, the least square-based method (PLS-SEM) was used to analyze the data rather than other structural equation modeling procedures such as covariance-based structural equation modeling (CB-SEM). PLS-SEM is mainly composed of two parts. The measurement model, also known as the outer model, is the first part. The outer model calculates each indicator's contribution to represent its associated latent variable and evaluates how well the combined indicators represent a construct. The inner model assesses the latent variables' direct and indirect relationships (Hair et al., 2016). Despite its popularity in social and behavioral sciences for multivariate data analysis, the SEM's application in academic learning has recently increased. SEM combines various data analysis methods to simultaneously examine the relationship between latent and observed variables. SEM is beneficial in understanding phenomena that cannot be observed directly, such as abilities, characteristics, intentions, attitudes, and perceptions in education research. SEM is a combination of factor and regression analysis.

## Demographic and Descriptive Analysis

In demographic analysis, the distribution of respondents based on gender illustrates that male respondents have dominated response rate with 55.3% (*n* = 207) compared to 44.7% (*n* = 167) who are female. Regarding marital status, it was observed that most of the respondents were single as 81.6% (305) compared to the 9.1% (*n* = 34) which were single, and a small number of respondents were committed and divorced as 8.8 and 0.5%, respectively. As for age was concerned, this study illustrates that majority of the respondents fall within the age 18–22 years as 68.7% (*n* = 257), the second-highest age group was 23–26, about 16.6% (*n* = 62) and 11.0% (*n* = 41) respondents were in the age bracket of the 27–31 years, and remaining 3.7% (*n* = 14) were between the age of 32–35. Moreover, it was also observed that over 69.5% (*n* = 260) of the responses came from the respondent having the undergraduate degree, 15.8% (*n* = 59) respondents were graduates, and 12.3% (*n* = 46) of the respondents have postgraduate qualification. The remaining 2.4% (*n* = 9) respondents have secondary-level qualifications. Moreover, the family structure of the respondents was also observed, the dominant family structure of the respondents was Nuclear/Separate Family 60.2% (*n* = 225), and the remaining were living in the joint family system, as reported by 39.8% (*n* = 149). It was also observed that 48.7% (*n* = 182) of the responses came from the respondent who had a monthly income less than PKR 20,000, and 23.5% (*n* = 88) respondent's monthly earnings were above PKR 50,000, and 13.1% (*n* = 49) of the respondent's income was PKR 20,000–30,000. The remaining 7.8% (*n* = 29) of respondents' earnings were between PKR 31,000 and 40,000 as shown in Table 1.

**Table 1:** Demographic statistics of respondents.

**Demographic variables**	**Category**	**Frequency**	**Percentage**
Gender	Male	207	55.3%
	Female	167	44.7%
Marital status	Single	305	81.6%
	Committed	33	8.8%
	Married	34	9.1%
	Divorced	2	0.5%
Age group	18–22	257	68.7%
	23–26	62	16.6%
	27–31	41	11.0%
	32–35	14	3.7%
Education	Secondary level	9	2.4%
	Undergraduate	260	69.5%
	Graduate	59	15.8%
	Postgraduate	46	12.3%
Family structure	Joint family	149	39.8%
	Nuclear/separate family	225	60.2%
Monthly income	Less than PKR 20,000	182	48.7%
	PKR 20,000–30,000	49	13.1%
	PKR 31,000–40,000	29	7.8%
	PKR 41,000–50,000	26	7.0%
	PKR above 50,000	88	23.5%

*n = 374*.

### Descriptive Statistics

This section will look at the descriptive statistic for the latent variable in this study: means and standard deviations of the latent constructs in the study. Means and standard deviations for the latent variables in the study were computed as a numerical summary of the data set. These variables were assessed using a five-point Likert scale ranging from 1 “strongly disagree” to 5 “strongly agree.” Table 2 shows the overall outcomes of latent constructs in terms of mean and standard deviation. The latent variable subjective wellbeing (SWB) has the highest mean of 3.32 and standard deviation of 0.98, indicating that most respondents rate SWB highly. Mindfulness (MIN) has the lowest mean of 2.54 and standard deviation of 0.86, indicating that MIN is rated highly by a small number of respondents. As a result, hopefulness (HOPE) and gratitude (GRA) have mean values of 2.84 and 2.83, respectively, with standard deviations of 0.97 and 0.99, demonstrating the respondents' given ratings, as shown in Table 2.

**Table 2:** Descriptive statistics.

**Variable**	**N**	**Minimum**	**Maximum**	**Mean**	**Std. Deviation**
Mindfulness	374	1.20	4.80	2.5452	0.86800
Gratitude	374	1.00	5.00	2.8378	0.99574
Subjective wellbeing	374	1.17	4.58	3.3223	0.98421
Hopefulness	374	1.00	5.00	2.8416	0.97475

### Measurement (Inner) Model Results

Before accessing the study's measurement (inner) model, a preliminary analysis was performed to ensure the accuracy of the data. This preliminary analysis includes the system's normality checks, missing value evaluations, outliers check, and standard method variance tests. However, some missing values were discovered and resolved using the most recommended mean replacement method. Researchers have widely anticipated this mean replacement method as the most effective tool for social science studies (Hair et al., 2017). This method is also unlikely to be affected by removing or changing other sample entries, such as lists of elimination and pairwise deletion, or by adjusting the mean values of the other variables (Hair et al., 2014).

**Figure 2:**
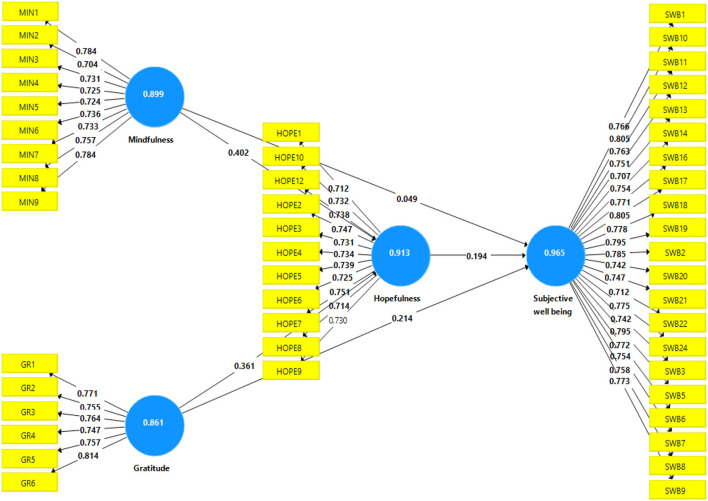
Measurement model. GRA, gratitude; MIN, mindfulness; HOPE, hopefulness; SWB, subjective wellbeing.

To evaluate the measurement model in the SEM, confirmatory factor analysis (CFA) was used to confirm and refine the items (components) and constructs (latent variables). First, we evaluated the measurement model for analyzing the relevance of indicator loadings of a construct in PLS-SEM analysis (Figure 2). The reliability test confirms a measurement instrument's consistency in measuring a specific construct, whereas the validity test demonstrates a respective instrument's ability to measure the construct that it claims to measure (Sekaran and Bougie, 2010). Second, the relationship between latent and observable constructs was established in the outer model. This step required examining three criteria: internal consistency reliability, convergent validity, and discriminant validity (Hair et al., 2016).

#### Internal Consistency Reliability

The Cronbach's alpha (CA) value was used to assess internal consistency among the components of each construct. Table 3 shows that the CA values of all constructs ranged from 0.86 to 0.96, and all values were more significant than the threshold value of 0.70, as suggested by Nunnally (1978). Furthermore, the composite reliability (CR) values for all constructs ranged from 0.89 to 0.96, higher than the recommended value of 0.70 (Nunnally and Bernstein, 1994). These tests ensured the internal consistency of these constructs, as shown in Table 3.

**Table 3:** Factor loading, Cronbach alpha, composite reliability, and AVE of the latent constructs.

**Variable**	**Item**	**Loading**	**CA**	**CR**	**AVE**	**Items deleted**
Gratitude	GR1	0.771	0.861	0.896	0.590	
	GR2	0.755				
	GR3	0.764				
	GR4	0.747				
	GR5	0.757				
	GR6	0.814				
Hopefulness	HOPE1	0.747	0.913	0.927	0.536	1
	HOPE2	0.731				
	HOPE3	0.734				
	HOPE4	0.739				
	HOPE5	0.725				
	HOPE6	0.751				
	HOPE7	0.714				
	HOPE8	0.730				
	HOPE9	0.747				
	HOPE10	0.732				
	HOPE12	0.738				
Mindfulness	MIN1	0.784	0.899	0.917	0.551	1
	MIN2	0.704				
	MIN3	0.731				
	MIN4	0.725				
	MIN5	0.724				
	MIN6	0.736				
	MIN7	0.733				
	MIN8	0.757				
	MIN9	0.784				
Subjective wellbeing	SWB1	0.766	0.965	0.967	0.585	3
	SWB2	0.785				
	SWB3	0.742				
	SWB5	0.795				
	SWB6	0.772				
	SWB7	0.754				
	SWB8	0.758				
	SWB9	0.773				
	SWB10	0.805				
	SWB11	0.763				
	SWB12	0.751				
	SWB13	0.707				
	SWB14	0.754				
	SWB16	0.771				
	SWB17	0.805				
	SWB18	0.778				
	SWB19	0.795				
	SWB20	0.742				
	SWB21	0.747				
	SWB22	0.712				
	SWB24	0.775				

*GRA, gratitude; MIN, mindfulness; HOPE, hopefulness; SWB, subjective wellbeing*.

#### Convergent Validity

Convergent validity shows the degree to which two assumed relate appear to be related even after the analysis. According to Hair et al. (2014), average variance extracted (AVE) is frequently used for determining a study's convergent validity. Therefore, primarily, we analyzed the item loadings for which 0.70 or above is the acceptable level for adequate item loadings (Fornell and Larcker, 1981). Thus, we obtained above 0.70 for all items, and four items were removed due to lower factor loading, as shown in Table 3. Moreover, Fornell and Larcker (1981) recommended AVE > 0.50 as an acceptable level for the research studies. The AVE values ranging from 0.53 to 0.59 were obtained as the minimum acceptable criteria, which show that convergent validity is established in this research.

#### Discriminant Validity

The (Fornell and Larcker, 1981) criterion was used to determine discriminant validity which explains how discriminately a construct varies from other constructs (Bagozzi and Yi, 1988). As a result, the square root of each construct's AVE value was calculated and compared to other constructs' cross-loading values. When compared to other correlation values, the square root of AVE for each construct had the highest value, showing a relationship with other components. Furthermore, construct correlations show that the variance shared between constructs is less than the variance shared by a construct with its indicators (see Table 4). Other criteria, such as the “heterotrait-monotrait ratio” of correlations (HTMT), were also employed to confirm discriminant validity as suggested by Henseler et al. (2015). The HTMT values show that the inter-construct ratios are <0.85, and the confidence intervals do not include a value of 1.0. As shown in Table 5, all latent constructs achieved the required discriminant validity. Therefore, the discriminant validity assessment of the constructs met the criteria. In conclusion, the measurement model's evaluations fulfilled the requirements, verifying the measurement model's applicability in this study.

**Table 4:** Discriminant validity (Fornell and Larcker).

	**GRA**	**HOPE**	**MIN**	**SWB**
GRA	0.768			
HOPE	0.534	0.732		
MIN	0.431	0.557	0.742	
SWB	0.338	0.335	0.249	0.765

*GRA, gratitude; MIN, mindfulness; HOPE, hopefulness; SWB, subjective wellbeing*.

**Table 5:** Discriminant validity (HTMT).

	**GRA**	**HOPE**	**MIN**	**SWB**
GRA				
HOPE	0.597			
MIN	0.477	0.598		
SWB	0.359	0.347	0.260	

*GRA, gratitude; MIN, mindfulness; HOPE, hopefulness; SWB, subjective wellbeing*.

### Structural Model Evaluation and Hypothesis Testing

#### Model Fit Tests

The SRMR (“standardized root means square residual”), d-ULS (“the squared Euclidean distance”), d-G (“the geodesic distance”), and the “Normed Fit Index” were used to evaluate the PLS-SEM model fit in this study (NFI). This study proved that the study achieved the model fitness criteria well, such as SRMR = 0.071 and 0.570. As the study shows, the SRMR value was not more than 0.08 (Richter et al., 2016), and the NFI value was not <0.8 (Hu and Bentler, 1998), indicating that the structural model satisfied the requirement.

#### Path Relationship Evaluations

Following the estimation of the outer model, the inner model is evaluated, which includes testing the hypotheses that have been proposed. As a result, in this study, we used a bootstrapping process with Smart PLS 3.0 and 500 samples to determine the significance of the path coefficients for testing the hypothesized relationships. According to Chin (2010), 200–1,000 bootstrapping samples provide reasonable estimates for standard errors. The regression coefficients (ß) were used to assess the direct and indirect relationships between constructs. In addition, depending on the *t*-value, the bootstrap technique was utilized to establish the importance of the ß values of direct and indirect correlations between constructs.

#### Direct Relationship Results

**Figure 3:**
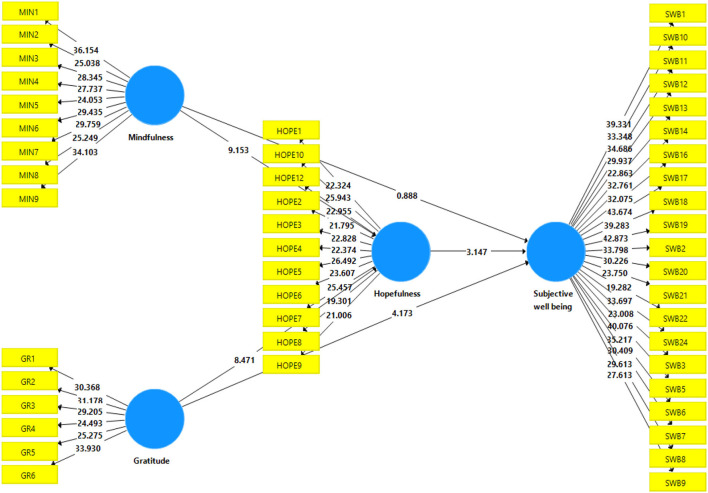
Structural model. GRA, gratitude; MIN, mindfulness; HOPE, hopefulness; SWB, subjective wellbeing.

Table 6 and Figure 3 shows that four out of five proposed hypotheses presenting direct relationships among constructs were significant at 5%. The results show that the relationship between gratitude and mindfulness significantly influences the individual's hopefulness; for instance, H1 (ß GRA -> HOPE = 0.361, *t* = 8.471, *p* = 0.000) and H2 (ß MIN -> HOPE = 0.402, *t* = 9.153, *p* = 0.000). This shows that if an individual is grateful and mindful, this leads the individual toward hopefulness. The H1 and H2 are in line with the previous research (Watson and Naragon, 2009; Datu and King, 2016), which also indicated that gratitude and mindfulness significantly influence hopefulness. Subsequently, direct relationships between gratitude, mindfulness, hopefulness, and subjective wellbeing (H4 and H5) were tested. The results revealed a significant association between gratitude, hopefulness, and subjective wellbeing, and H3 and H5 are accepted, as the results indicated H3 (ß GRA -> SWB = 0.214, *t* = 4.173, *p* = 0.000) and H5 (ß EM -> EI = 0.194, *t* = 3.147, *p* = 0.002). The results show that gratitude and hopefulness directly impact subjective wellbeing as claimed by previous studies such as Bailey and Snyder (2007) and Feng and Yin (2021). However, the H4 was not found statistically significant as ß MIN -> SWB = 0.049, *t* = 0.888, *p* = 0.375, which shows that mindfulness does not influence subjective wellbeing. The detailed results are shown in Table 6.

**Table 6:** Direct relationship results.

**H**	**Path**	**Beta**	**STDEV**	**T statistics**	***P*-values**	**Decision**
H1	GRA -> HOPE	0.361	0.043	8.471	0.000	Accepted
H2	MIN -> HOPE	0.402	0.044	9.153	0.000	Accepted
H3	GRA -> SWB	0.214	0.051	4.173	0.000	Accepted
H4	MIN -> SWB	0.049	0.055	0.888	0.375	Not accepted
H5	HOPE -> SWB	0.194	0.062	3.147	0.002	Accepted

*GRA, gratitude; MIN, mindfulness; HOPE, hopefulness; SWB, subjective wellbeing*.

#### Indirect Relationships

About the indirect (mediation) results, this study revealed that the relationship between gratitude and subjective wellbeing is significantly mediated by the hopefulness as (ß GRA -> HOPE -> SWB = 0.070, *t*-value = 3.056, and *p*-value = 0.002). In the same vein, the relationship between mindfulness and subjective wellbeing is significantly mediated by the hopefulness as ß MIN -> HOPE -> SWB = 0.078, *t*-value = 2.865, and *p*-value = 0.004, which confirms that H6 and H7 are statistically significant and accepted. Moreover, the results of the H7 reported complete mediation as the direct relationship between mindfulness and subjective wellbeing was not accepted (see Table 6). This means that if an individual is more hopeful, then his mindfulness and subjective wellbeing nexus would be stronger which is not found as direct. The brief results of H6 and H7 are shown in Table 7.

**Table 7:** Mediation results.

**H**	**Path**	**Beta**	**STDEV**	**T statistics**	***P*-values**	**Decision**
H6	GRA -> HOPE -> SWB	0.070	0.023	3.056	0.002	Accepted
H7	MIN -> HOPE -> SWB	0.078	0.027	2.865	0.004	Accepted

*GRA, gratitude; MIN, mindfulness; HOPE, hopefulness; SWB, subjective wellbeing*.

### Predictive Capability Evaluation

An essential component of evaluating a SEM is testing the model's prediction accuracy (PA) and predictive relevance (PR). Because it shows the amount of variance explained by each endogenous variable, the coefficient of determination (R2 value) has been used to assess PA (Hair et al., 2019). According to Hair et al. (2019), a value of R2 ranging between 0 and 1 indicated a higher level of PA, with a significant value of R2 indicating a greater level of PA. As shown in Table 8, the R2 values of the latent constructs hopefulness were 0.417, demonstrating a significant level of PA, while the R2 value of the other latent variable, subjective wellbeing, was 0.150, indicating a sufficient level of predictive accuracy (Henseler et al., 2015). The evaluation of predictive relevance (PR), on the other hand, was assessed by running the blindfolding procedure to compute the Q2 value. According to Hair et al. (2014), a >0 Q2 value shows that the PR of the latent constructs is achieved. This study result revealed that the Q2 values generated for each construct are significant, implying that the proposed model in this study has good PR. Table 8 shows that R-square and Q2 values were found to be sufficient by the defined criteria.

**Table 8:** R-square and Q2 of latent exogenous variables.

**Exogenous variables**	**R Square**	***Q^2^***
Hopefulness	0.417	0.220
Subjective wellbeing	0.150	0.083

## Discussion

Mindfulness and gratitude are the positive virtues that many religious and secular intellectuals have promoted for centuries. However, at present time, social researchers are also finding scientific evidence of mindfulness and gratitude as practical tools to increase the wellbeing of humanity (Lambert et al., 2010; Meherunissa, 2016; Alkozei et al., 2018). This research study found evidence of a positive effect of gratitude on mental wellbeing in current work. Furthermore, we found evidence of the mechanism of hopefulness through which gratitude relates to mental wellbeing. Surprisingly, this study did not find evidence of a direct impact of mindfulness on the wellbeing of the sample set. However, by introducing hopefulness as a mediator, analysis shows a significant relationship between mindfulness and wellbeing.

This study supports the broaden-and-build theory (Fredrickson, 2001). The theory states that positive emotions expand present thought–action repertoires, which broaden the display of thoughts that come to mind (Fredrickson and Branigan, 2005). This theory talks about the upward spiral of broadening and building. The results of this study affirmed this idea showing that one positive emotion, that is, gratitude, leads to another positive emotion creating hopefulness in the youth's mind and that increases overall subjective wellbeing.

One critical result of this study is that mindfulness does not have a significant direct impact on the wellbeing of participants, whereas previous studies have provided evidence of significant effects of mindfulness state on mental wellbeing (Keng et al., 2011; Tang et al., 2019; Allen et al., 2021). However, when hopefulness is added as a mediating variable in the relationship between mindfulness and wellbeing in this study, the relationship between both variables becomes significant. If this unique finding is reflected keeping in mind the cultural context of the participants of this study, then it makes quite a sense that if youth are only encouraged to remain mindful of the present situation, it might be possible that they being conscious of prevailing injustice, lack of opportunities available on merit, too many daily hassles such as heavy traffics and non-compliance of a traffic rule or general desensitization from the positivity of people around may make them feel helpless. According to Maslow's hierarchy of needs, this helplessness may keep their focus on survival needs or, according to Maslow's hierarchy of needs (1980), at lower-order needs. This study highlights the importance of cultivating hopefulness in young youth while teaching mindfulness techniques. It can be expected to enhance hopefulness that may enhance the sense of responsibility and autonomy in youth. These positive emotions are expected to motivate youth to consider oneself as change agents and make collaborative efforts in a positive direction. In summary, it feels secure to suggest that the increasing level of hopefulness in Pakistani youth might increase the wellbeing of youth.

### Theoretical and Social Implication

This study has contributed to both theoretical and social implications. According to existing literature, mindfulness has a direct impact on wellbeing; however, in this research, it was found that hopefulness plays a mediating role between mindfulness and wellbeing, which is the new contribution to present literature specifically in the Pakistani context. Therefore, this study is an essential contribution to validating the broaden-and-build theory's (Fredrickson, 2001) applicability on diversified cultures. This study also highlights that youth suffering from depression or other psychological issues can benefit from positive psychological interventions. As hopelessness is a significant factor of a depressive state, it can be indicated that inducing hopefulness could be a significant element of the treatment plan of professional clinical psychologists. However, we recommend further research to investigate clinical samples and examine the duration of the effects.

The significant social implication of this study in introducing hopefulness is one way of improving the social wellbeing of Pakistani youth. Even the present government is highly involved in motivating youth to participate in nation building, this study can be used as a guideline for the government to continue designing youth empowerment programs at local, regional, national, or even collaboration with international agencies. It may escalate hopefulness in youth for a better future.

### Limitations and Future Directions

There are a few limitations of this study. This study used the cross-sectional method, so the generalizability of findings can be questioned. Furthermore, the theory used in the study focuses on youth only; similar studies with other age groups can highlight the importance of cultivating positive emotions. Therefore, there are some crucial recommendations for future research on this topic. First, as the broaden-and-build theory postulates that peoples' experiences of constructive emotions broaden their momentary thought–action repertoires, which results in the accumulation of physical, intellectual, psychological, and social resources, this study only studied and found evidence of increment of psychological resources named as increased life satisfaction, positive emotions and decreased negative emotions, etc. There is room for more research to see the impact of positive emotions on physical, intellectual, and social resources. Second, research studies exploring behavior modifications through positive emotions can also add to the literature on practical implications of broaden-and-build theory. Third, the trait of hopefulness needs to be explored more about other positive emotions such as happiness and grit. Fourth, it is essential to note that we explored wellbeing using the BBC subjective wellbeing scale (BBC-SWB), which measured many psychological variables such as autonomy, life satisfaction, and positive relationships with others named as few cumulatively calling it subjective wellbeing. Studying the impact of positive emotions on these variables separately will enhance the understanding of subjective wellbeing in a more specific way. Fifth, different sets of samples such as comparing working and non-working women or studying the effect of positive emotions at the workplace on the wellbeing of employees can help to prepare specific human resource policies for different industries. Sixth, there is a high need to design experimental studies to show the real effects of positive emotions in this study: gratitude and mindfulness on subjective wellbeing. Such studies using experimental methods can also promote experiential learning of the general population to understand the influential role of positive emotions in leading a happy and meaningful life. Seventh, to understand hopefulness as a mechanism of the relationship between mindfulness and subjective wellbeing in developing nations, it is essential to replicate this study on diverse populations of other developing nations. Lastly, in this study, we have used PLS_SEM for the analysis, which has some limitations. For instance, it does not provide a global fit statistic for models. This is a particular limitation and becomes difficult when an overall fit statistic is not available. For the future research, researchers may use other statistical approaches to gain more insight about the topic.

In conclusion, it can be said that the advantages of nurturing positive emotions on subjective wellbeing are the main highlights of this study. This study also signifies the importance of studying the emotion of hopefulness in the Pakistani context in more depth. It is proved to be an essential mechanism of strengthening the relationship between other positive emotions and the subjective wellbeing of youth.

## Data Availability Statement

The original contributions presented in the study are included in the article/supplementary material, further inquiries can be directed to the corresponding author/s.

## Ethics Statement

Ethical review and approval was not required for the study on human participants in accordance with the local legislation and institutional requirements. The patients/participants provided their written informed consent to participate in this study.

## Author Contributions

SA has taken the overall responsibilities of the manuscript and she gives the idea of the issue to be investigated. MJA has written the Introduction part. IR worked on literature Review section of the manuscript and had taken the responsibility of Data Collection. MA helped in the methodology part and run the statistical analysis. KK has compiled the discussion part and he has provided a technical support throughout the manuscript. All authors contributed to the article and approved the submitted version.

## Conflict of Interest

The authors declare that the research was conducted in the absence of any commercial or financial relationships that could be construed as a potential conflict of interest.

## Publisher's Note

All claims expressed in this article are solely those of the authors and do not necessarily represent those of their affiliated organizations, or those of the publisher, the editors and the reviewers. Any product that may be evaluated in this article, or claim that may be made by its manufacturer, is not guaranteed or endorsed by the publisher.
